# Differential Effects of Two Herbivore-Induced Plant Volatiles on the Oviposition of *Chilo suppressalis*

**DOI:** 10.3390/plants14152384

**Published:** 2025-08-02

**Authors:** Xiaowei Yang, Chang Liu, Xixi Jia, Chen Zhang, Lanzhi Han, Wanlun Cai, Yunhe Li

**Affiliations:** 1 Hubei Insect Resources Utilization and Sustainable Pest Management Key Laboratory, College of Plant Science and Technology, Huazhong Agricultural University, Wuhan 430070, China; 2 State Key Laboratory for Biology of Plant Diseases and Insect Pests, Institute of Plant Protection, Chinese Academy of Agricultural Sciences, Beijing 100193, China; yangxiaowei01@caas.cn (X.Y.); 15075372498@163.com (C.Z.); 3 State Key Laboratory of Crop Stress Adaptation and Improvement, State Key Laboratory of Cotton Bio-Breeding and Integrated Utilization, School of Life Sciences, Henan University, Kaifeng 475004, China

**Keywords:** *Chilo suppressalis*, herbivore-induced plant volatiles, reproduction, oviposition behavior, biocontrol

## Abstract

Herbivore-induced plant volatiles (HIPVs) are well known for their roles in herbivore deterrence and attraction of natural enemies, but their direct impact on insect reproduction remains largely unexplored. In this study, we provide novel evidence that two representative HIPVs, 2-heptanol and α-cedrene, exert opposing effects on the reproduction of *Chilo suppressalis*, a major rice pest. While both volatiles repelled adults, α-cedrene unexpectedly enhanced oviposition, whereas 2-heptanol significantly suppressed egg laying. To examine these effects, we conducted oviposition assays, preoviposition and longevity tests, combined with qPCR and transcriptome analyses to explore underlying molecular responses. Mechanistically, α-cedrene upregulated *Kr-h1*, a gene linked to juvenile hormone signaling and vitellogenesis, promoting reproductive investment. Transcriptomic profiling revealed divergent molecular responses: α-cedrene activated reproductive pathways, whereas 2-heptanol induced stress- and immune-related genes, suggesting a trade-off between stress defense and reproduction. These findings demonstrate that HIPVs can exert compound-specific reproductive effects beyond repellency. This work fills a key knowledge gap and highlights the potential of HIPVs as precision tools in pest management strategies that exploit behavioral and physiological vulnerabilities beyond repellency.

## 1. Introduction

Plant volatiles are pivotal mediators in the co-evolution between plants and insects, functioning in deterring herbivores, attracting natural enemies and so on [1,2]. Among them, herbivore-induced plant volatiles (HIPVs) represent a key defensive strategy, helping plants to resist insect attacks [3,4,5]. For instance, several caterpillar-induced volatiles are highly repellent to female moths [6]. However, these volatiles can also be exploited by insect pests for host location, oviposition, and other behaviors, highlighting the dynamic interplay between plant defense and insect adaptation [3].

Although HIPVs have been widely studied for their roles in feeding behavior, locomotion, and predator attraction, their influence on insect reproductive processes remains poorly understood. HIPVs could paradoxically serve as oviposition cues. The herbivore-induced cotton volatile (E)-4,8-dimethyl-1,3,7-nonatriene (DMNT) suppresses mating and oviposition in *Spodoptera littoralis* [7]. Conversely, the corn-derived HIPV component (Z)-3-hexenyl acetate stimulates oviposition preference in female *Spodoptera frugiperda* [8], demonstrating how insects exploit plant signals to their advantage. These contrasting examples underscore the nuanced role of HIPVs in shaping insect reproductive behavior, positioning them as promising targets for precision pest control strategies.

Given the underexplored link between HIPVs and insect reproduction, a closer examination of reproductive processes is warranted. Insect reproduction is a pivotal driver of pest population dynamics and outbreak severity [9,10]. Consequently, insect reproductive processes have been a primary focus for the development of novel biocontrol methods [9,11,12,13]. Reproduction encompasses a complex sequence of events, including reproductive organ development, gametogenesis, mating, and oviposition [14,15]. Even minor disruptions to these processes can significantly reduce oviposition efficiency [15]. For example, exposure to sublethal doses of insecticides can impair the development of reproductive organs, leading to a reduction in egg-laying capacity, even in the absence of visible harm to the insect’s general health [16]. Alterations in environmental conditions, such as temperature fluctuations, can disrupt mating behaviors, resulting in lower fertilization rates and fewer eggs being produced [17]. This complexity is amplified by insects’ sophisticated molecular perception systems, whose mechanisms for detecting volatile compounds to modulate reproduction remain poorly understood [18]. Different HIPV components can modulate insect reproduction in both positive and negative ways, offering valuable research opportunities. Understanding these mechanisms could lead to the development of HIPV-based reproductive inhibition strategies, providing an innovative, environmentally sustainable approach to pest management.

To explore the ecological relevance of HIPV-mediated reproductive regulation, we selected the rice stem borer, *Chilo suppressalis* Walker (*Lepidoptera*: *Pyralidae*), as our model species. This pest is one of the most destructive insects affecting rice cultivation in Asia, causing substantial yield losses through larval tunneling that disrupts nutrient transport and damages vascular tissues. It is also known for inducing characteristic symptoms such as “dead hearts” and “white ears” in rice plants [19,20,21]. In addition, *C. suppressalis* has strong reliance on olfactory cues to locate suitable host plants for oviposition. This makes the adult stage particularly vulnerable to disruption by HIPVs. Manipulating such cues offers a promising strategy to interfere with reproduction, thereby preventing larval infestation before it occurs. Moreover, due to extensive insecticide use, *C. suppressalis* populations have developed resistance to many chemical pesticides [22,23], underscoring the need for green pest control technologies. HIPVs, as natural signaling molecules, provide a feasible avenue to disrupt reproduction through subtle modulation of host selection and oviposition behavior. Therefore, *C. suppressalis* represents both a practical and mechanistically relevant model for investigating HIPV-mediated reproductive regulation in pest management. In this study, we investigated the impact of two HIPV compounds, 2-heptanol and α-cedrene, on the reproductive biology of *C. suppressalis*.

## 2. Results

### 2.1. 2-Heptanol and α-Cedrene Differentially Regulate Oviposition

The HIPVs 2-heptanol and α-cedrene exhibit repellent properties and affect the reproductive performance of *C. suppressalis*. We investigated the effects of different concentrations of these volatiles on adult fecundity, preoviposition period, and longevity.

**Figure 1:**
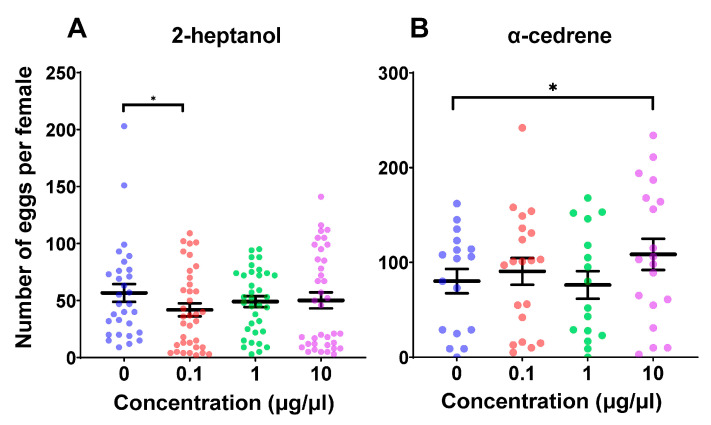
Effect of 2-heptanol and α-cedrene on fecundity in *Chilo suppressalis*. The number of eggs laid by female adults of *C. suppressalis* when treated with different concentrations of 2-heptanol (**A**) and α-cedrene (**B**). Each individual dot signifies a replicate sample. The mean ± SE of the data is depicted in the graph. Asterisks (*) indicate statistically significant differences (*p* < 0.05) between various treatment groups.

Female fecundity showed no significant overall differences among the 2-heptanol-treated groups using a generalized linear mixed model (GLMM) (*χ*^2^  = 5.17, *df*  = 3, *p* = 0.160). However, an exploratory pairwise comparison indicated a potential reduction in fecundity at the 0.1 μg/μL concentration compared to the control group (*p* = 0.038) (Figure 1A). Given the exploratory nature of this study, we retained this result to highlight a potential trend that may warrant further investigation. Meanwhile, female fecundity showed significant differences among the α-cedrene-treated groups (GLMM, *χ*^2^  = 9.31, *df*  = 3, *p* = 0.025), and the fecundity increased when exposed to a concentration of 10 μg/μL α-cedrene compared to the control group (*p* = 0.046) (Figure 1B).

### 2.2. Volatile-Induced Changes in Preoviposition Period and Adult Longevity

**Figure 2:**
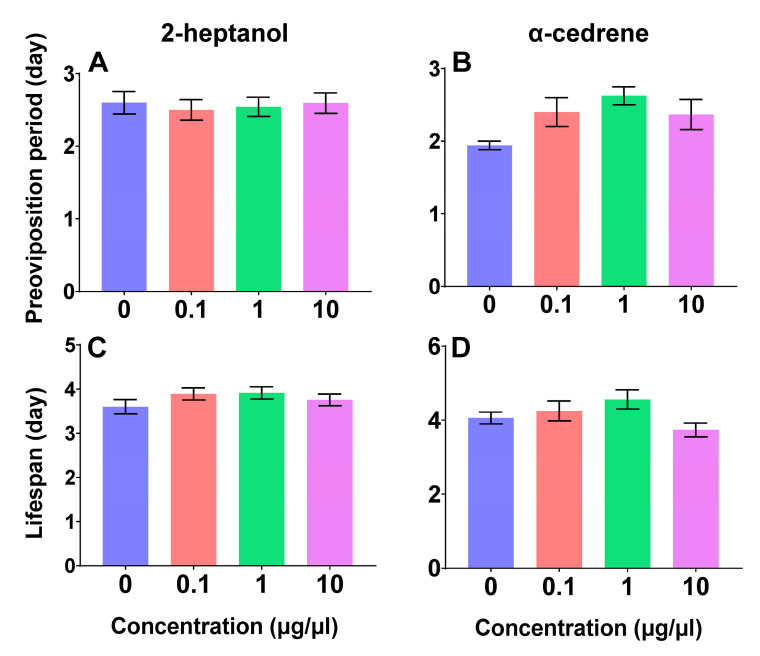
The preoviposition period of *C. suppressalis* treated with 2-heptanol (**A**) and α-cedrene (**B**). The longevity of *C. suppressalis* treated with 2-heptanol (**C**) and α-cedrene (**D**). The data are shown as the mean ± SE. One-way ANOVA was used, and post hoc analyses using Tukey’s Honestly Significant Difference (HSD) test were conducted for pairwise comparisons between groups.

To assess the potential direct effects on oviposition timing or indirect effects on fecundity through altered adult survival, we examined the preoviposition period and adult lifespan of *C. suppressalis* exposed to these two compounds. The preoviposition period exhibited no significant differences when subjected to treatments with 2-heptanol (*F*_3,134_ = 0.11, *p* = 0.95) (Figure 2A) and α-cedrene (*F*_3,68_ = 2.73, *p* = 0.05) (Figure 2B). Similarly, the adult lifespan demonstrated no significant variations in response to 2-heptanol (*F*_3,134_ = 1.379, *p* = 0.25) (Figure 2C) and α-cedrene (*F*_3,68_ = 2.291, *p* = 0.09) (Figure 2D).

### 2.3. The Expression Levels of Vitellogenin (Vg) in the Fat Body and Krüppel Homolog 1 (Kr-h1) in the Ovaries

**Figure 3:**
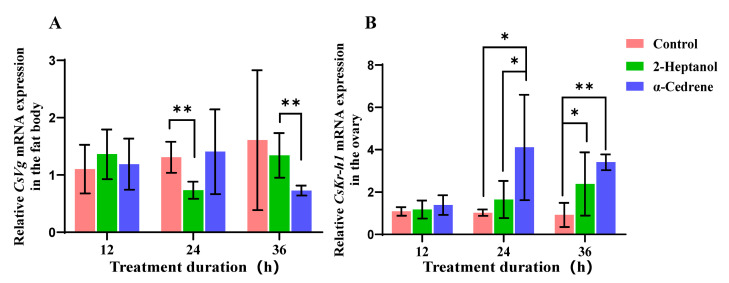
The expression pattern of the *Vg* gene in the fat body (**A**) and the *Kr-h1* gene in the ovary (**B**) of *C. suppressalis* after volatile treatment. The data are shown as the mean ± SE. The differences between groups were analyzed using Student’s *t*-tests. * represents *p* < 0.05, and ** means *p* < 0.01. N = 3.

A significant downregulation of the *Vg* gene was observed in the fat body of *C. suppressalis* 24 h after treatment with 2-heptanol (Figure 3A, *p* < 0.01). In contrast, *Vg* expression did not significantly differ between the cedrol-treated and control groups at this time point. At 36 h post-treatment, *Vg* expression in the 2-heptanol group was significantly higher than that in the cedrol group, while no significant differences were detected between either treatment group or the control.

A significant upregulation of the *Kr-h1* gene was observed in the ovary of *C. suppressalis* at both 24 h (*p* < 0.05) and 36 h (*p* < 0.01) after treatment with α-cedrene (Figure 3B). Additionally, treatment with 2-heptanol also led to a significant upregulation of *Kr-h1* gene in the ovary of *C. suppressalis* at 36 h (*p* < 0.05) post-treatment (*p* < 0.05; Figure 3B).

### 2.4. Transcriptome Analysis of C. suppressalis After Volatile Treatment

**Figure 4:**
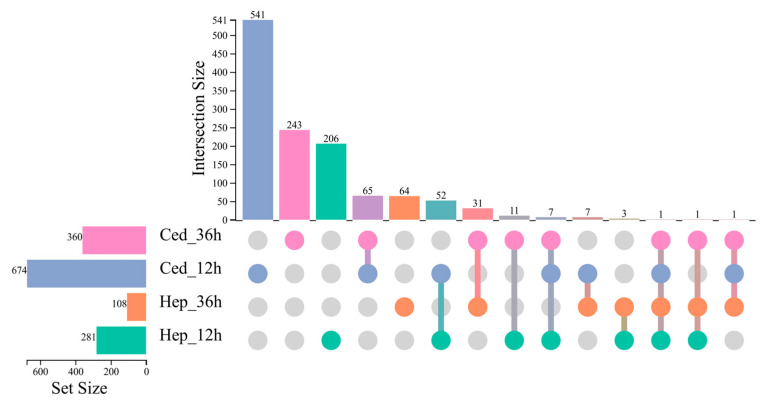
UpSet Venn plot showing the overlap of differentially expressed genes (DEGs) among treatment groups. Each treatment group is represented by a sample name: Hep_12h: 2-heptanol treatment for 12 h; Hep_36h: 2-heptanol treatment for 36 h; Ced_12h: α-cedrene treatment for 12 h; Ced_36h: α-cedrene treatment for 36 h. The horizontal bars on the lower left indicate the total number of DEGs identified in each treatment group relative to its respective control (n-hexane at 12 h or 36 h, respectively). The vertical bars at the top represent the number of DEGs that are shared exclusively by the treatment groups, which are connected by filled color dots below, but those not present in any other group are indicated by unconnected gray dots.

The transcriptome sequencing data was obtained from the Sequence Read Archive of the National Center for Biotechnology Information (NCBI) under Bioproject PRJNA1280920. The sequencing data were of high quality and suitable for downstream analyses (Figure S1), with 88.16% to 93.25% of reads from all samples successfully mapped to the reference genome (Figure S2). At 12 h post-treatment with the volatiles, a total of 281 differentially expressed genes (DEGs) were identified between the 2-heptanol treatment group and the control group, while 674 DEGs were identified between the α-cedrene treatment group and the control group (Figure S3), with 52 common genes shared uniquely between the two comparisons (Figure 4). Among these co-regulated genes, *Csup000932*, encoding the chorion transcription factor Cf2, may be involved in the early formation of the insect egg chamber [24]. Other DEGs included *novel00305*, encoding cathepsin B, which is implicated in multiple reproductive functions [25,26,27,28], such as gametogenesis [25] and vitellogenin metabolism [26]. CYP4M38, a cytochrome P450 enzyme, is known to function in hormone metabolism and detoxification processes, potentially influencing reproductive development [29,30].

At 36 h post-treatment, 108 DEGs were identified between the 2-heptanol treatment group and the control group, and 360 DEGs were identified between the α-cedrene treatment group and the control group (Figure S3), with 31 common genes shared between the two comparisons (Figure 4). Among them, *Csup003211*, encoding carboxypeptidase, is involved in digestion, immune defense, and development, while *Csup006304*, encoding apolipophorin III, plays a role in lipid transport and may be associated with egg-laying or hatching success [31].

Only one gene was found to be differentially expressed across all four comparisons. This gene, *Csup008876*, encodes ribulose-phosphate 3-epimerase, a key enzyme in the pentose phosphate pathway (PPP).

**Figure 5:**
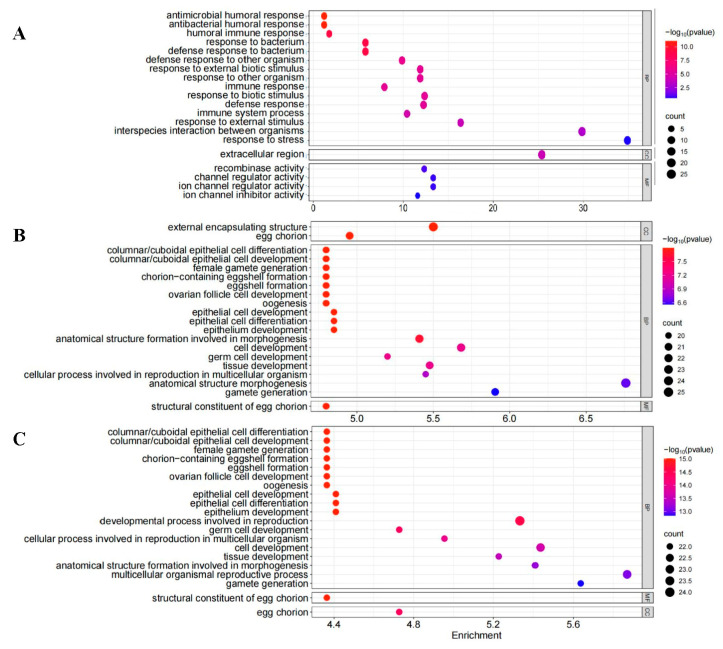
Top 20 GO terms for GO enrichment analysis of differential genes treated with 2-heptanol for 12 h (**A**), α-cedrene for 12h (**B**) and α-cedrene for 36 h (**C**). The *Y*-axis represents GO function classification; the X-axis represents the enrichment multiple of differential genes; the size of the scatter represents the number of genes enriched in the pathway; scatter color representation logarithmic transformation of *p*-value.

Further analysis showed that α-cedrene significantly upregulated genes associated with reproduction and hormone biosynthesis, whereas 2-heptanol treatment predominantly induced genes involved in stress and immune responses, including those related to antimicrobial defense (Figure 5). After 12 h of 2-heptanol exposure treatment, GO enrichment analysis revealed that DEGs were predominantly concentrated in the following immune-related categories: antimicrobial humoral response (GO:0019730), antibacterial humoral response (GO:0019731), humoral immune response (GO:0006959), response to bacterium (GO:0009617) and so on (Figure 5A). No category showed significant enrichment 36 h after treatment with 2-heptanol in *C. suppressalis*.

In contrast, GO enrichment analysis revealed that after 12 h of α-cedrene exposure treatment in female *C. suppressalis* moths, DEGs were enriched in reproduction-related categories: external encapsulating structure (GO:0030312), egg chorion (GO:0042600), structural components of the egg chorion (GO:0005213), female gamete generation (GO:0007292), and so on (Figure 5B).

Similar to the 12 h exposure to α-cedrene, the DEGs after 36 h of α-cedrene exposure were also enriched in reproduction-related GO categories (Figure 5C), such as structural components of the egg chorion (GO:0005213), female gamete generation (GO:0007292), formation of eggshells with chorion (GO:0007304), and eggshell formation (GO:0030703).

Several genes related to immune response and reproduction were selected for qPCR to verify their expression patterns (Figures S1–S4). The results showed that the expression patterns were consistent with the transcriptome data.

## 3. Discussion

Current studies often highlight the role of HIPVs as herbivore deterrents [5,32], attractants for natural enemies [33], or alarm signals for neighboring plants [4,34]. Additionally, some studies have explored the role of HIPVs in insect oviposition behavior, treating HIPV components as cues for either oviposition or oviposition deterrence [35,36,37]. For example, certain plant volatiles have been reported to exhibit physiological activity and attract gravid females [35,38], suggesting that some HIPVs may serve as oviposition attractants rather than deterrents. However, in most cases, reduced pest oviposition after exposure to HIPVs was considered as part of deterrence. Yet, deterrence and oviposition may not always be directly correlated. Our findings indicate that HIPV functions are more context-dependent and complex than the simple categorizations.

A previous study showed that both 2-heptanol and α-cedrene are repellents for *C. suppressalis* [32]. This study investigated their specific impacts on reproductive performance. Notably, while both volatiles repelled *C. suppressalis* adults, α-cedrene uniquely enhanced reproductive behavior (Figure 1). It is important to note that a broader statistical perspective was adopted in this exploratory study to identify potentially meaningful trends. While overall model significance was not always achieved, post hoc comparisons revealed possible compound-specific effects on oviposition counts following 2-heptanol treatment. These results are intended as hypothesis-generating rather than confirmatory, and should be interpreted with caution pending validation in future studies. Similar to 2-heptanol, general repellents such as DEET have also been reported to negatively affect oviposition in gravid female mosquitoes, likely by interfering with olfactory cues important for oviposition site selection [39]. However, it is rare for repellent compounds to stimulate oviposition, as observed with α-cedrene. These results underscore the need for a more nuanced understanding of HIPV functions beyond broad-spectrum repellency.

Neither 2-heptanol nor α-cedrene significantly affected the overall adult lifespan and preoviposition period of *C. suppressalis* across treatment concentrations (Figure 2). Since adult lifespan remained unchanged, the observed changes in oviposition behavior are unlikely to be confounded by differences in survival. These results suggest that the two volatiles exert direct and compound-specific influences on reproductive, rather than indirectly affecting it through alterations in adult survival. It is worth noting, however, that in other insect systems and environmental or chemical factors can influence reproduction through lifespan modulation. For example, low barometric pressure was found to accelerate ovarian development and shorten the preoviposition period in *Mythimna separata*, although it also reduced female lifespan [40]. Similarly, in *Drosophila melanogaster*, lipid metabolism and fat storage dynamics have been shown to mediate trade-offs between fecundity and lifespan [41]. 2-heptanol exhibited no measurable effect on the preoviposition period, whereas α-cedrene did. This suggests that α-cedrene may interfere with the initiation of oviposition through reproductive timing.

The contrasting effects of 2-heptanol and α-cedrene on fecundity align with distinct molecular pathways. Gene expression analysis revealed that α-cedrene consistently upregulated *Kr-h1*, a key transcription factor in the JH signaling cascade, at both 24 and 36 h post-treatment (Figure 3). Since JH plays a central role in regulating vitellogenesis and oocyte maturation in insects [9,42], the sustained upregulation of *Kr-h1* in the ovaries suggests that α-cedrene may enhance reproductive output by promoting JH-dependent pathways. In contrast, 2-heptanol induced a significant downregulation of *Vg* expression in the fat body at 24 h, with no significant difference observed at 36 h (Figure 3), indicating a transient suppression of vitellogenin synthesis. Although a late upregulation of Kr-h1 was observed in the ovaries at 36 h post-2-heptanol exposure, the temporal mismatch with *Vg* expression and the absence of a sustained response may underlie its weaker effect on reproduction. Taken together, these results suggest that both HIPVs can modulate JH signaling in the ovary, but α-cedrene induces a more rapid and sustained activation of this pathway, while 2-heptanol exerts only a transient regulatory effect, particularly on early processes.

Transcriptomic analysis revealed distinct molecular responses to the two HIPV compounds. At 12 h post-treatment, both 2-heptanol and α-cedrene triggered significant transcriptional changes, with α-cedrene eliciting a greater number of DEGs. The transcriptomic differences observed between the two treatments suggest divergent physiological impacts of 2-heptanol and α-cedrene on female reproductive function. The upregulation of reproduction- and hormone-related genes in response to α-cedrene indicates a promotive effect on reproductive physiology. In contrast, 2-heptanol exposure triggered the activation of immune- and stress-related genes, implying that it may act as a mild physiological stressor. Specifically, α-cedrene upregulated genes associated with reproduction and hormone biosynthesis, suggesting a promotive effect on reproductive physiology. In contrast, 2-heptanol primarily induced genes related to stress and immune responses, including those involved in antimicrobial defense (Figure 5). These results imply that 2-heptanol may function as a mild physiological stressor, activating immune-related pathways that potentially divert metabolic resources away from reproduction—a mechanism commonly observed in insect–plant interactions. The induction of insect immune responses by plant-derived volatiles has also been documented in other studies. For instance, the melon fruit fly exhibited an immune response when fed a diet containing nerolidol, a plant-derived sesquiterpene [43]. Similarly, Cis-3-hexen-1-ol, an important component in maize HIPVs, induced the detoxification enzymes and gene expression in *S. frugiperda and S. litura* [44]. These studies support the hypothesis that certain volatiles can shift insect physiological priorities toward defense rather than reproduction, consistent with the transcriptomic patterns observed in our study.

GO enrichment analysis after 12 h of 2-heptanol treatment revealed an unexpected enrichment of immune-related pathways—particularly those linked to bacterial defense—in female *C. suppressalis* moths, rather than pathways associated with reproduction (Figure 5A). This finding suggests that 2-heptanol may disrupt immune homeostasis, indirectly impairing reproductive processes. Conversely, GO analysis following 12 h α-cedrene exposure showed that α-cedrene predominantly influenced pathways involved in early egg formation and reproductive biosynthesis (Figure 5B). Notably, DEGs in these categories exhibited significant upregulation, indicating a stimulatory effect of α-cedrene on female moth reproduction.

While this study focused on the direct impact of HIPVs on *C. suppressalis* reproduction, it is important to consider the broader ecological implications. Potential non-target effects—such as impacts on natural enemies (e.g., parasitoids or predators) or symbiotic microbiota—were not evaluated in this study. Future research should examine whether 2-heptanol affects beneficial organisms in agroecosystems. For field application, HIPVs like 2-heptanol could be formulated into slow-release dispensers and used in ‘push–pull’ strategies, or integrated with pheromone traps to optimize pest deterrence in a sustainable and targeted manner.

The divergent effects of these HIPVs offer unique opportunities for pest control. The oviposition-suppressing activity of 2-heptanol positions it as a strong candidate for integrated pest management (IPM), where it could be deployed to deter pests from target crops and reduce infestation rates. Conversely, despite its repellent properties, the fecundity-enhancing effect of α-cedrene limits its practical application in pest control, as it may boost pest populations. Furthermore, breeding rice varieties that constitutively emit 2-heptanol or engineering synthetic blends to disrupt pest reproduction could reduce reliance on chemical insecticides, mitigating resistance issues.

## 4. Materials and Methods

### 4.1. The Rice Stem Borer Strain

The rice stem borer, *C. suppressalis,* population used in this study has been maintained on an artificial diet under controlled laboratory conditions for an extended period, as described in previous study [45]. The rearing conditions were set at 27 ± 1 °C with a relative humidity of 75% and a photoperiod of 16L:8D. Newly emerged *C. suppressalis* adults were collected every 12 h and provided with a 10% honey solution. The age of the adults was recorded starting from the day of eclosion, designated as day 0, and continued accordingly for subsequent days.

### 4.2. Decoy Treatment

The lure core used in the experiment consisted of a natural vulcanized rubber plug with dimensions of 11.5 mm in upper diameter, 6.5 mm in lower diameter, and 15 mm in height (Keyun Biocontrol, Jiyuan, China). The blank lure core was pre-soaked in n-hexane (Sigma-Aldrich, St. Louis, MO, USA), and then dried to eliminate any residual odors. The tested reagents, 2-heptanol and α-cedrene (Sigma-Aldrich, St. Louis, MO, USA), were diluted in n-hexane, and 100 μL of the designated concentration of volatile solution was applied to the lure core until fully absorbed.

### 4.3. Effects of Volatiles on Fecundity and Longevity of Female Adults

A newly emerged male and female of *C. suppressalis* were placed together in a 700 mL plastic cup. To provide food, a cotton ball soaked in a 10% honey solution was added to the cup. Additionally, a lure core, pre-soaked with either the control solution (n-hexane) or one of the test reagents (2-heptanol or α-cedrene) at specific concentrations, was also placed in the cup. The experiment included n-hexane as the control, along with three concentrations each of 2-heptanol (0.1, 1.0, and 10.0 μg/μL) and α-cedrene (0.1, 1.0, and 10.0 μg/μL). The cup was then covered with a mesh cloth to prevent the insects from flying away. The photoperiod, temperature and humidity were consistent with the rearing conditions described above. The survival of female moths and the number of eggs were observed twice a day (9:00 am and 9:00 pm), and the total oviposition of female moths throughout their lifetime was recorded, as well as the preoviposition period and longevity. This experiment was repeated twice, with 15–30 couples in each repetition.

### 4.4. Sample Collection for High-Throughput Sequencing and qPCR

According to the results of the experiment above, 2-heptanol (0.1 μg/μL) and α-cedrene (10.0 μg/μL) were selected for the following experiment. Newly emerged *C. suppressalis* adults were collected daily and transferred into individual 700 mL plastic cups. Inside each cup, a lure core loaded with the tested reagent (either n-hexane as a control, 0.1 μg/μL of 2-heptanol, or 10.0 μg/μL of α-cedrene) and a cotton ball containing 10% honey were also placed. Then an adult male and an adult female were placed in a cup, which was subsequently covered with mesh cloth.

Female *C. suppressalis* moths were collected at 12 h and 36 h post-treatment for transcriptome sequencing. Following a wash with 75% ethanol once and PBS twice, the whole bodies were frozen using liquid nitrogen and stored at −80 °C. Ten female moths were used as a replicate, and three replicates were established and collected at each time point for each treatment condition.

Female *C. suppressalis* moths in the cup were dissected at 12 h, 24 h and 36 h post-treatment for qPCR analysis. Following a wash with 75% ethanol once and PBS twice, the ovaries and fat bodies were obtained and stored at −80 °C. Five females were combined as a replicate. A total of three replicates were established and collected at each time point for each treatment condition.

### 4.5. RNA Extraction and qPCR for Vg and Kr-h1 Gene Expression

The total RNA of the tissue samples was extracted using the RNAprep pure tissue kit (Tiangen, Beijing, China) according to the manufacturer’s protocol. A NanoDrop spectrophotometer and 1% agarose gel electrophoresis were used to detect the RNA quality and integrity. A 1 μg amount of total RNA was reverse-transcribed using the TransScrip One-Step gDNA Removal and cDNA Synthesis SuperMix (TransGen Biotech, Beijing, China) following the manufacturer’s protocol.

*Vg* is a crucial gene involved in insect oviposition, and previous studies have demonstrated that *Vg* expression is often regulated by *Kr-h1*, a key transcription factor in the juvenile hormone (JH) signaling pathway. Therefore, we examined the expression levels of both *Vg* and *Kr-h1* in female adults after treatment with these two compounds by quantitative real-time PCR (qPCR) with the gene-specific primers (Table S4). The qPCR was performed with the Perfectstart Green qPCR Supermix (TransGen Biotech, Beijing, China). qPCR reactions of serial dilutions of cDNA samples were used to determine the amplification efficiency for each pair of primers to ensure the amplification efficiency within the range of 90–110%. The relative gene expression levels were calculated using the 2^−ΔΔCT^ method, using the expression of the elongation factor 1 alpha (EF1) gene as the internal control.

### 4.6. Transcriptome Sequencing and Bioinformatics Analysis

For transcriptome sequencing, total RNA was extracted from the whole body of striped rice stemborers using Trizol reagent (Tiangen, Beijing, China). RNA quality and integrity were assessed using a NanoDrop 2000 (Thermo Fisher Scientific, Wilmington, DE, USA) and an Agilent 2100 Bioanalyzer (Agilent Technologies, Santa Clara, CA, USA). RNA samples were then sent to Allwegene (Beijing, China) for library construction, sequencing and data analysis. Poly (A) + mRNA was enriched using magnetic beads conjugated with Oligo (dT), followed by mRNA fragmentation. First-strand cDNA synthesis was performed using random hexamer primers, and the second-strand cDNA synthesis was carried out. After purification, end-repair, A-tailing, and adapter ligation were performed on the double-stranded cDNA. Size selection for cDNA fragments ranging from 200 to 250 bp was conducted using AMPure XP magnetic beads (Beckman Coulter Life Sciences, Indianapolis, IN, USA). PCR amplification was performed to construct the cDNA library. Following quality control, pooling was conducted based on the library’s effective concentration and desired data output. Sequencing was performed on an Illumina NovaSeq 6000 platform (Illumina, San Diego, CA, USA) using a paired-end 150 (PE150) strategy.

Raw sequencing data were filtered with Trimmomatic (v0.33) by removing reads with adapters, more than 10% ambiguous nucleotides (N), or over 50% low-quality bases (Q ≤ 20). The filtered reads were aligned to the reference genome of *C. suppressalis* (available at http://v2.insect-genome.com/Organism/177 (accessed on 6 October 2024)) using STAR (v2.5.2b). The alignment results were assembled using Cufflinks (v2.1.1), and novel genes were identified, too. Gene expression levels for each sample were analyzed using HTSeq (v0.5.4 p3) with the “union” mode. Differential expression analysis was performed using DESeq (v1.10.1) [46], and *p*-values were adjusted for multiple testing using the Benjamini–Hochberg method to control the false discovery rate (FDR). Genes with a *p*-value < 0.05 were considered significantly differentially expressed. GO enrichment analysis was performed by Goseq (v1.22) [47].

### 4.7. Data Analysis

Statistical analyses were performed using R version 3.6.1 (R Core Team, www.r-project.org (accessed on 6 October 2024)). Oviposition data were analyzed using a GLMM with Poisson distribution (lme4 package version 1.1-14) to account for overdispersion, and model fitness was assessed using likelihood ratio tests (LRTs). Differences in preoviposition period and lifespan were evaluated using one-way ANOVA, and post hoc analyses using Tukey’s Honestly Significant Difference (HSD) test were conducted for pairwise comparisons between groups. Gene expression differences were analyzed using Student’s *t*-tests.

## Data Availability

The reference genome of *C. suppressalis* is available at http://v2.insect-genome.com/Organism/177 (accessed on 6 October 2024). The sequencing data are available in the Sequence Read Archive of the NCBI under Bioproject PRJNA1280920.

## Supplementary Materials

The following supporting information can be downloaded at https://www.mdpi.com/article/10.3390/plants14152384/s1. Figure S1: Verification of transcriptome differential genes of the female moths of *C. suppressalis* treated with α-cedarene for 12 h; Figure S2. Verification of transcriptome differential genes of the female moths of *C. suppressalis* treated with α-cedarene for 36 h; Figure S3. Verification of transcriptome differential genes of the female moths of *C. suppressalis* treated with 2-heptanol for 12 h; Figure S4. Verification of transcriptome differential genes of the female moths of *C. suppressalis* treated with 2-heptanol for 36 h; Table S1. Summary of transcriptome sequencing data quality; Table S2. Summary of clean reads mapped to reference genome for *C. suppressalis*; Table S3. Differentially expressed genes (DEGs) between each treatment group and its corresponding control group; Table S4. Primers for quantitative real-time PCR (qPCR).

## Abbreviations

The following abbreviations are used in this manuscript:

HIPV	Herbivore-induced plant volatile
GLMM	Generalized linear mixed model
DEG	Differentially expressed gene
