# Supplementary material for: Differential Effects of Two Herbivore-Induced Plant Volatiles on the Oviposition of Chilo suppressalis
Source: Plants (Basel). 2025 Aug 2;14(15):2384. doi: 10.3390/plants14152384 (PMC12349140; doi:10.3390/plants14152384)
Supplement: Supplementary file 1 [file plants-14-02384-s001.zip › Supplementary Figures.pptx]

## Slide 1
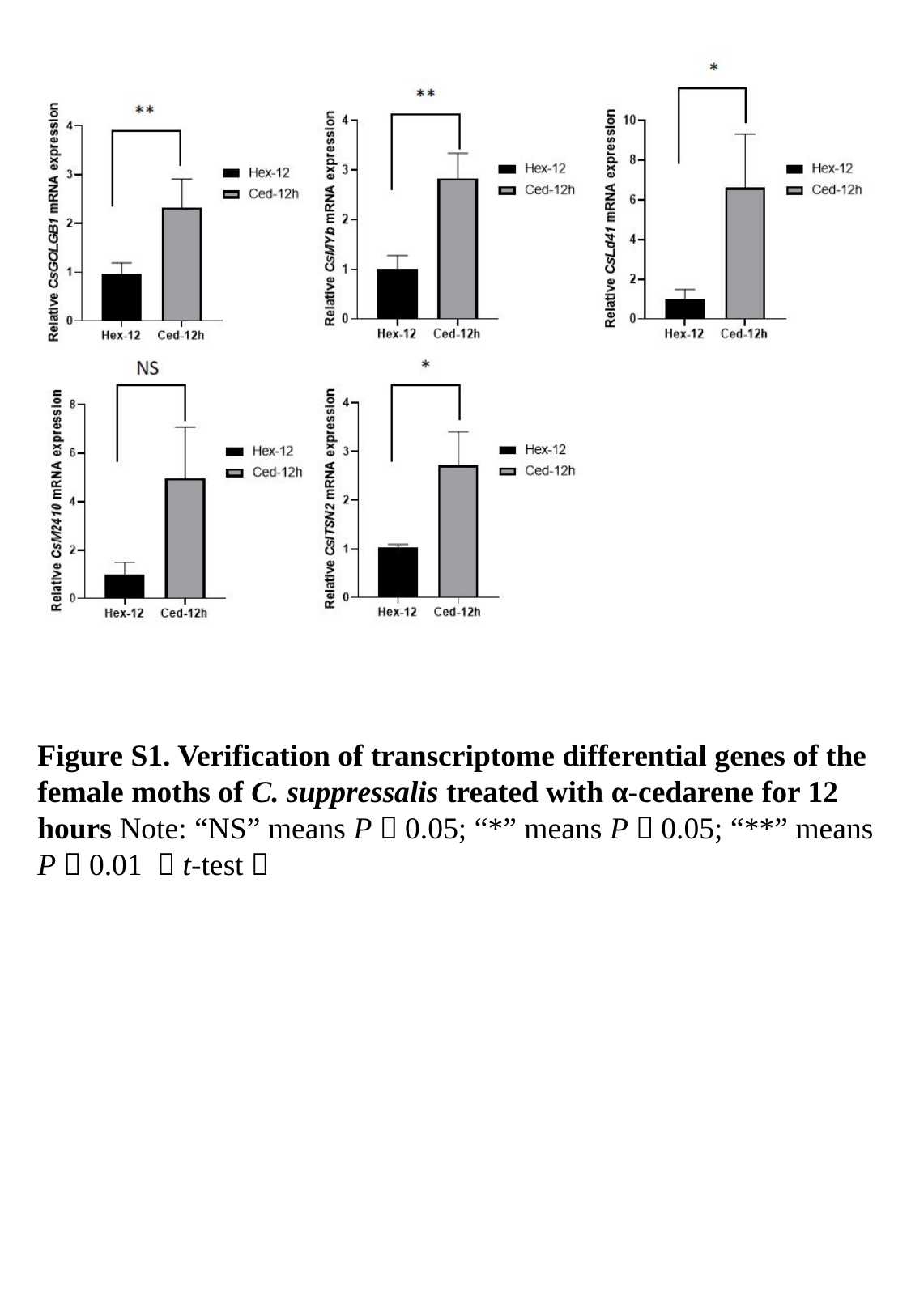

Figure S1. Verification of transcriptome differential genes of the female moths of C. suppressalis treated with α-cedarene for 12 hours Note: “NS” means P＞0.05; “*” means P＜0.05; “**” means P＜0.01 （t-test）

## Slide 2
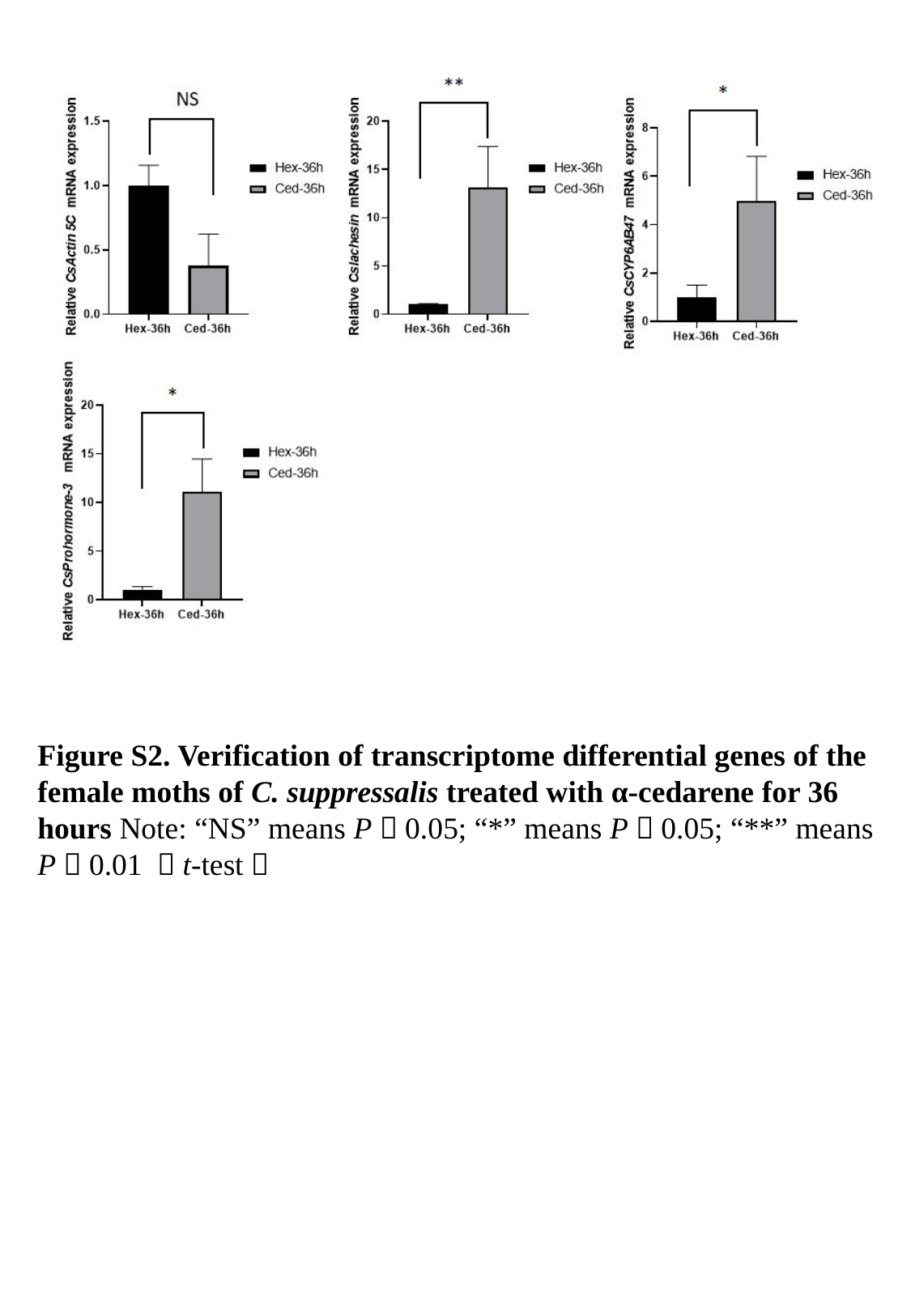

Figure S2. Verification of transcriptome differential genes of the female moths of C. suppressalis treated with α-cedarene for 36 hours Note: “NS” means P＞0.05; “*” means P＜0.05; “**” means P＜0.01 （t-test）

## Slide 3
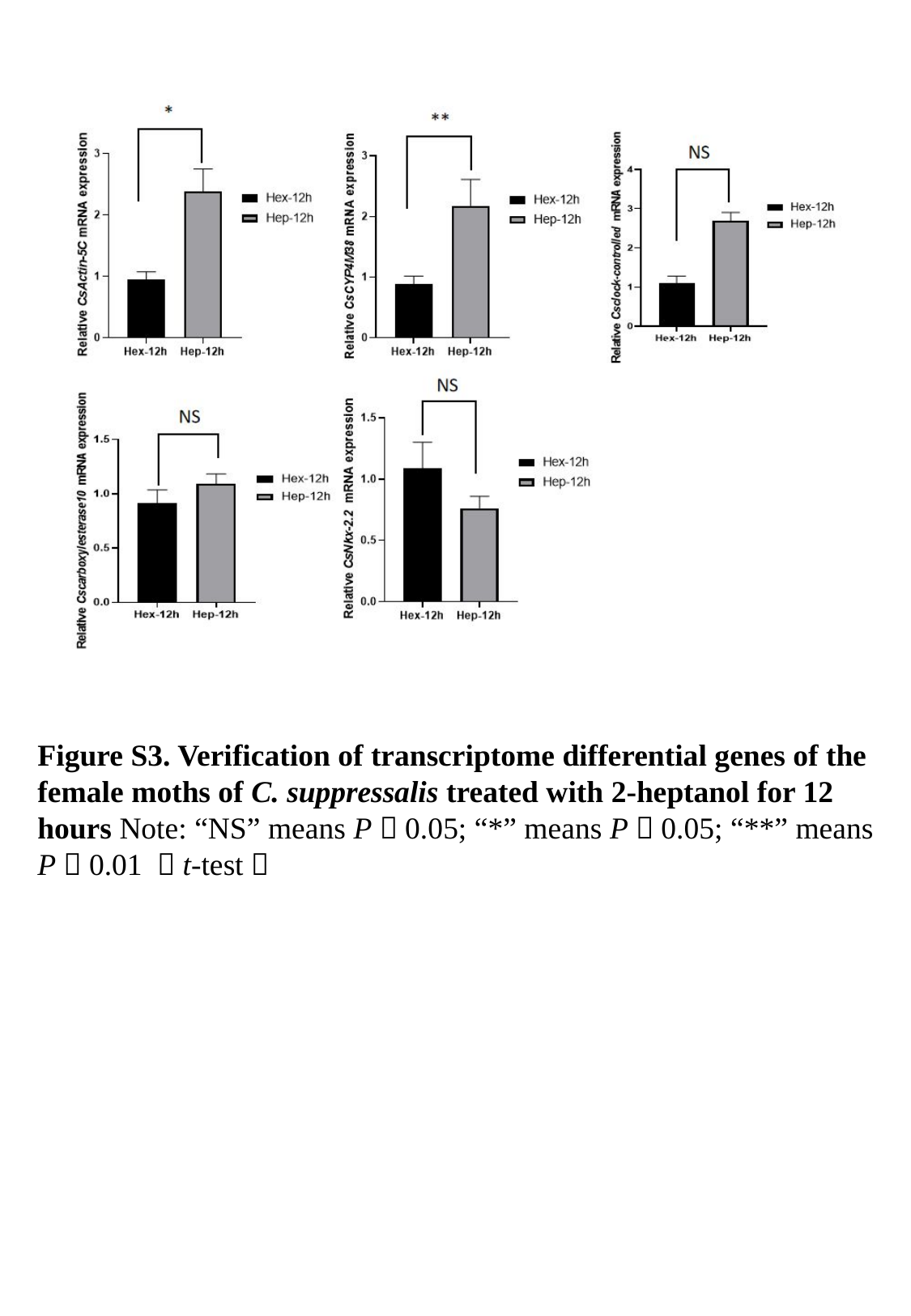

Figure S3. Verification of transcriptome differential genes of the female moths of C. suppressalis treated with 2-heptanol for 12 hours Note: “NS” means P＞0.05; “*” means P＜0.05; “**” means P＜0.01 （t-test）

## Slide 4
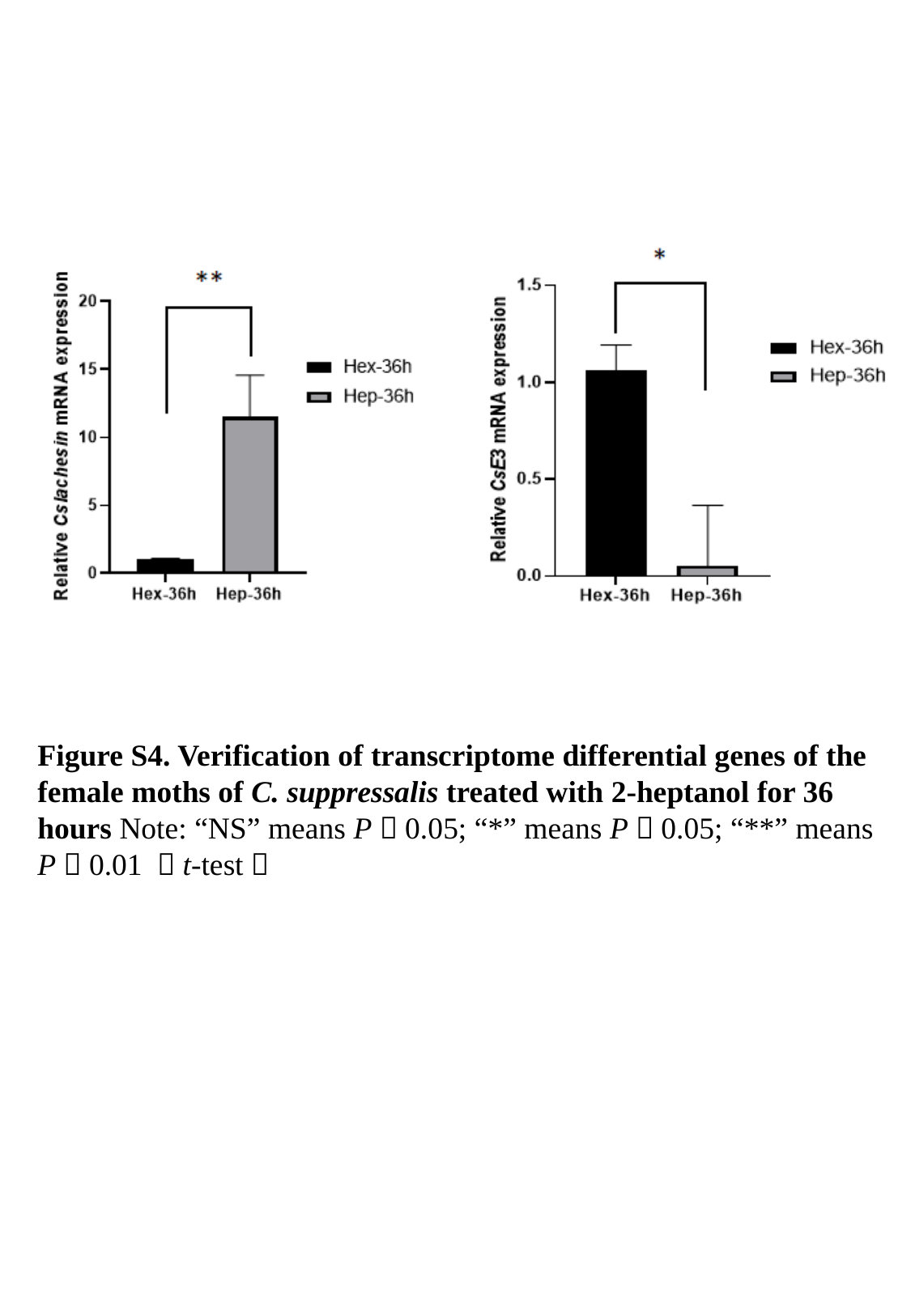

Figure S4. Verification of transcriptome differential genes of the female moths of C. suppressalis treated with 2-heptanol for 36 hours Note: “NS” means P＞0.05; “*” means P＜0.05; “**” means P＜0.01 （t-test）
